# 8-Meth­oxy-4-(4-methoxy­phen­yl)quinoline

**DOI:** 10.1107/S1600536809052623

**Published:** 2009-12-12

**Authors:** Ligia Llovera, Teresa González, Pavel Anzenbacher, Simón E. López

**Affiliations:** a Laboratorio 223, Departamento de Química, Universidad Simón Bolívar (USB), Apartado 89000, Caracas 1080-A, Venezuela; b Centro de Química, Instituto Venezolano de Investigaciones Científicas (IVIC), Apartado 21827, Caracas 1020-A, Venezuela; c Department of Chemistry, Center for Photochemical Sciences, Bowling Green State University (BGSU), Bowling Green, OH 43-403, USA.

## Abstract

In the title compound, C_17_H_15_NO_2_, the dihedral angle between the quinoline and benzene ring systems is 62.17 (1)°. In the crystal, zigzag chains propagating in *c* are linked by C—H⋯O hydrogen bonds, and weak C—H⋯π inter­actions link the chains.

## Related literature

The title compound was prepared as an inter­mediate for the synthesis of aluminium(III) quinolinolate complexes, which are important for their semiconductor properties and as electron-transport layer materials in organic light-emitting devices (OLEDs) (Montes *et al.*, 2006 ▶). For related literature, see: Dienys *et al.* (1977 ▶); Muscia *et al.* (2006 ▶); Pérez-Bolívar *et al.* (2006 ▶).
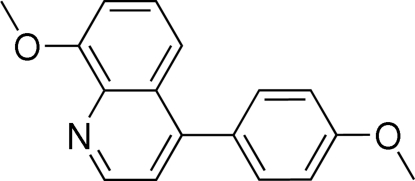


## Experimental

### 

#### Crystal data

C_17_H_15_NO_2_
*M* *_r_* = 265.30
Monoclinic, 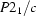

*a* = 9.362 (2) Å
*b* = 10.355 (2) Å
*c* = 14.276 (4) Å
β = 101.556 (6)°
*V* = 1355.9 (5) Å^3^
*Z* = 4
Mo *K*α radiation
μ = 0.09 mm^−1^
*T* = 295 K
0.45 × 0.42 × 0.40 mm

#### Data collection

Rigaku AFC-7S Mercury diffractometer
Absorption correction: multi-scan (*ABSCOR*; Jacobson, 1998 ▶) *T*_min_ = 0.940, *T*_max_ = 0.980
15093 measured reflections
2785 independent reflections
1868 reflections with *I* > 2σ(*I*)
*R*_int_ = 0.037
Standard reflections: 0

#### Refinement

*R*[*F* ^2^ > 2σ(*F* ^2^)] = 0.057
*wR*(*F* ^2^) = 0.145
*S* = 1.12
2785 reflections
181 parameters
H-atom parameters constrained
Δρ_max_ = 0.14 e Å^−3^
Δρ_min_ = −0.23 e Å^−3^

### 

Data collection: *CrystalClear* (Rigaku/MSC, 2005 ▶)); cell refinement: *CrystalClear*; data reduction: *CrystalClear*; program(s) used to solve structure: *SHELXTL* (Sheldrick, 2008 ▶); program(s) used to refine structure: *SHELXTL*; molecular graphics: *SHELXTL* and *DIAMOND* (Brandenburg, 1999 ▶); software used to prepare material for publication: *SHELXTL* and *PLATON* (Spek, 2009 ▶).

## Supplementary Material

Crystal structure: contains datablocks global, I. DOI: 10.1107/S1600536809052623/hb5272sup1.cif

Structure factors: contains datablocks I. DOI: 10.1107/S1600536809052623/hb5272Isup2.hkl

Additional supplementary materials:  crystallographic information; 3D view; checkCIF report

## Figures and Tables

**Table 1:** Hydrogen-bond geometry (Å, °)

*D*—H⋯*A*	*D*—H	H⋯*A*	*D*⋯*A*	*D*—H⋯*A*
C17—H17*A*⋯O1^i^	0.96	2.55	3.322 (3)	137
C2—H2*A*⋯*Cg*2^ii^	0.93	2.81	3.622 (2)	146
C6—H6*A*⋯*Cg*3^iii^	0.93	2.80	3.592 (2)	144
C17—H17*B*⋯*Cg*2^iv^	0.96	2.78	3.580 (3)	142

Symmetry codes: (i) 
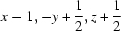
; (ii) 
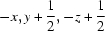
; (iii) 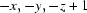; (iv) 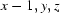. *Cg*2 and *Cg*3 are the centroids of the C4–C9 and C10–C14 rings, respectively.

**Fig. 1.**
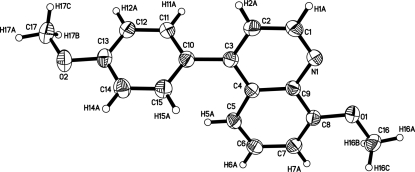
The molecular structure of (I), showing displacement elipsoids drawn at the 35% probability level and H atoms are shown as spheres of arbitrary radii.

## supplementary crystallographic information

### Comment

The title compound (I), was prepared as a valued intermediate for the synthesis of aluminium (III) quinolinolate complexes, important for their semiconductor properties and useful properties as electron-transport layer materials in organic light-emitting devices (OLEDs) (Montes *et al.*, 2006).

The molecular structure of (I) is shown in Figure 1 with their respective labels. All bond lenghts are in good agreement with the tabulated standard values (Table 1). In this structure quinoline motif is essentially planar (with a mean deviation 0.0213 Å), in which the maximum deviation is around atoms C1 (0.0277 Å) and C6 (0.0333 Å), respectively (see Figure 1). The methoxyphenyl substituent make a dihedral angle of 62.17 (1)° with respect to the quinoline group. Other strinking feature of (I) is that the plane defined by atoms contained for the metoxy groups C16/O1/C8/C9 and C17/O2/C13/C14 are almost co-planar with the phenyl rings with values of dihedral angle 1.73 (3) and 1.42 (2)°, respectively.

### Experimental

A solution of 3-dimethylamino-1-(4-methoxy-phenyl)-propan-1-one (300 mg, 1.45 mmol) and 2-methoxy-phenylamine (180 mg, 1.46 mmol) in ethanol (15 ml) was stirred at room temperature in a round bottom flask. After 15 minutes, concentrated hydrochloric acid (0.5 ml) was added dropwise and the mixture stirred under reflux for 8 h. The reaction mixture was cooled to room temperature and poured into an ice bath. The yellow solution was neutralized with a saturated solution of sodium bicarbonate to pH 7, extracted with ethyl acetate (20 ml × 3), and washed with water (20 ml × 3) and brine (10 ml × 2). The aqueous layer was extracted with dichloromethane (20 ml × 2). The organic layers were dried over anhydrous magnesium sulfate, filtered through cotton and the filtrate concentrated under vacuum to provide a reddish oil. The oil was purified by column chromatography on silica gel (mobile phase: ethyl acetate-hexane, 6:4) to afford a light-brown solid (100 mg, 26%). *M*.p. 146–147 °C; ^1^H NMR (500 MHz, CDCl_3_), d(p.p.m.): 3.82 (s, 3H), 4.05 (s, 3H), 6.99 (t, 3H, J= 8.5 Hz), 7.27 (d, ^1^H, J= 4.4 Hz), 7.34 (t, ^1^H, J= 8.2 Hz), 7.37 (d, 2H, J= 8.7 Hz), 7.47 (d, ^1^H, J= 8.6 Hz), 8.88 (d, ^1^H, J= 4.4 Hz). ^13^C NMR (126 MHz, CDCl_3_), d (p.p.m.): 55.29 (C17), 55.99 (C_16_), 107.21 (C_7_), 113.90 (C_12_ and C_14_), 117.59 (C_5_), 121.91 (C_6_), 126.37 (C_2_), 127.93 (C_4_), 130.48 (C_9_), 130.73 (_C11_ and C_15_), 140.67 (C_3_), 147.95 (C_10_), 148.68 (C_1_), 155.55 (C_8_), 159.72 (C_13_). IR (KBr, cm^-1^) 3004, 2934, 2838, 1673, 1607, 1501, 1249. EI—MS: m/*z* (%): 266 (100) [*M*^+^], 251 (40) [M—CH_3_^+^].

Light brown blocks of (I) were obtained by slow evaporation of dichloromethane/hexane

### Refinement

All H atoms were placed in geometrically idealized positions and constrained to ride on their parent atoms, with C—H distances of 0.93 (aromatic) and 0.96 Å (methyl) and with *U*_iso_(H) = 1.5 (1.2 for aromatic H atoms) times *U*_eq_(C).

### Crystal data

**Table:**

C_17_H_15_NO_2_	*F*(000) = 560
*M**_r_* = 265.30	*D*_x_ = 1.300 Mg m^−^^3^
Monoclinic, *P*2_1_/*c*	Mo *K*α radiation, λ = 0.71070 Å
Hall symbol: -P 2ybc	Cell parameters from 8153 reflections
*a* = 9.362 (2) Å	θ = 4.4–55.8°
*b* = 10.355 (2) Å	µ = 0.09 mm^−^^1^
*c* = 14.276 (4) Å	*T* = 295 K
β = 101.556 (6)°	Block, light brown
*V* = 1355.9 (5) Å^3^	0.45 × 0.42 × 0.40 mm
*Z* = 4	

### Data collection

**Table:**

Rigaku AFC-7S Mercury diffractometer	2785 independent reflections
Radiation source: Normal-focus sealed tube	1868 reflections with *I* > 2σ(*I*)
graphite	*R*_int_ = 0.037
ω scans	θ_max_ = 28.1°, θ_min_ = 56.1°
Absorption correction: multi-scan (*ABSCOR*; Jacobson, 1998)	*h* = −12→12
*T*_min_ = 0.940, *T*_max_ = 0.980	*k* = −13→13
15093 measured reflections	*l* = −18→18

### Refinement

**Table:**

Refinement on *F*^2^	Primary atom site location: structure-invariant direct methods
Least-squares matrix: full	Secondary atom site location: difference Fourier map
*R*[*F*^2^ > 2σ(*F*^2^)] = 0.057	Hydrogen site location: inferred from neighbouring sites
*wR*(*F*^2^) = 0.145	H-atom parameters constrained
*S* = 1.12	*w* = 1/[σ^2^(*F*_o_^2^) + (0.0538*P*)^2^ + 0.3325*P*] where *P* = (*F*_o_^2^ + 2*F*_c_^2^)/3
2785 reflections	(Δ/σ)_max_ < 0.001
181 parameters	Δρ_max_ = 0.14 e Å^−^^3^
0 restraints	Δρ_min_ = −0.23 e Å^−^^3^

### Special details

**Table:**

Geometry. All e.s.d.'s (except the e.s.d. in the dihedral angle between two l.s. planes) are estimated using the full covariance matrix. The cell e.s.d.'s are taken into account individually in the estimation of e.s.d.'s in distances, angles and torsion angles; correlations between e.s.d.'s in cell parameters are only used when they are defined by crystal symmetry. An approximate (isotropic) treatment of cell e.s.d.'s is used for estimating e.s.d.'s involving l.s. planes.
Refinement. Refinement of *F*^2^ against ALL reflections. The weighted *R*-factor *wR* and goodness of fit *S* are based on *F*^2^, conventional *R*-factors *R* are based on *F*, with *F* set to zero for negative *F*^2^. The threshold expression of *F*^2^ > σ(*F*^2^) is used only for calculating *R*-factors(gt) *etc*. and is not relevant to the choice of reflections for refinement. *R*-factors based on *F*^2^ are statistically about twice as large as those based on *F*, and *R*- factors based on ALL data will be even larger.

### Fractional atomic coordinates and isotropic or equivalent isotropic displacement parameters (Å^2^)

**Table:**

	*x*	*y*	*z*	*U*_iso_*/*U*_eq_	
O1	0.42442 (15)	0.09277 (15)	0.26653 (10)	0.0576 (4)	
O2	−0.41766 (17)	0.34353 (16)	0.58150 (11)	0.0644 (5)	
N1	0.22017 (18)	0.27365 (17)	0.23541 (12)	0.0507 (5)	
C1	0.1179 (2)	0.3624 (2)	0.22134 (16)	0.0554 (6)	
H1A	0.1163	0.4196	0.1709	0.066*	
C2	0.0111 (2)	0.3774 (2)	0.27655 (15)	0.0522 (5)	
H2A	−0.0583	0.4424	0.2618	0.063*	
C3	0.0082 (2)	0.29631 (19)	0.35232 (14)	0.0427 (5)	
C4	0.1152 (2)	0.19600 (18)	0.36953 (13)	0.0397 (5)	
C5	0.1192 (2)	0.10133 (19)	0.44199 (14)	0.0456 (5)	
H5A	0.0515	0.1042	0.4815	0.055*	
C6	0.2215 (2)	0.0066 (2)	0.45375 (15)	0.0493 (5)	
H6A	0.2217	−0.0556	0.5007	0.059*	
C7	0.3275 (2)	0.0006 (2)	0.39636 (15)	0.0495 (5)	
H7A	0.3978	−0.0641	0.4066	0.059*	
C8	0.3271 (2)	0.08987 (19)	0.32555 (14)	0.0441 (5)	
C9	0.2189 (2)	0.18964 (19)	0.30946 (13)	0.0414 (5)	
C10	−0.1047 (2)	0.31030 (19)	0.41186 (14)	0.0447 (5)	
C11	−0.2518 (2)	0.2989 (2)	0.37074 (15)	0.0504 (5)	
H11A	−0.2788	0.2841	0.3053	0.060*	
C12	−0.3598 (2)	0.3091 (2)	0.42425 (15)	0.0512 (5)	
H12A	−0.4574	0.3001	0.3950	0.061*	
C13	−0.3213 (2)	0.33263 (19)	0.52103 (16)	0.0491 (5)	
C14	−0.1750 (2)	0.3464 (2)	0.56353 (16)	0.0554 (6)	
H14A	−0.1487	0.3634	0.6287	0.066*	
C15	−0.0685 (2)	0.3350 (2)	0.50986 (15)	0.0524 (5)	
H15A	0.0289	0.3440	0.5394	0.063*	
C16	0.5389 (2)	−0.0015 (2)	0.28179 (17)	0.0632 (7)	
H16A	0.5999	0.0110	0.2359	0.095*	
H16B	0.4973	−0.0865	0.2745	0.095*	
H16C	0.5962	0.0080	0.3452	0.095*	
C17	−0.5693 (2)	0.3254 (2)	0.54239 (18)	0.0653 (7)	
H17A	−0.6242	0.3361	0.5919	0.098*	
H17B	−0.5847	0.2401	0.5161	0.098*	
H17C	−0.6007	0.3879	0.4929	0.098*	

### Atomic displacement parameters (Å^2^)

**Table:**

	*U*^11^	*U*^22^	*U*^33^	*U*^12^	*U*^13^	*U*^23^
O1	0.0497 (8)	0.0726 (10)	0.0547 (9)	0.0123 (7)	0.0206 (7)	0.0055 (8)
O2	0.0592 (10)	0.0763 (11)	0.0643 (10)	−0.0038 (8)	0.0281 (8)	−0.0167 (8)
N1	0.0478 (10)	0.0584 (11)	0.0476 (10)	0.0007 (9)	0.0135 (8)	0.0117 (9)
C1	0.0549 (13)	0.0598 (14)	0.0523 (13)	0.0004 (11)	0.0127 (11)	0.0194 (11)
C2	0.0478 (12)	0.0542 (13)	0.0549 (13)	0.0060 (10)	0.0113 (10)	0.0133 (10)
C3	0.0396 (10)	0.0438 (11)	0.0442 (11)	−0.0042 (9)	0.0074 (9)	0.0013 (9)
C4	0.0384 (10)	0.0414 (11)	0.0387 (10)	−0.0056 (8)	0.0067 (8)	−0.0004 (8)
C5	0.0474 (11)	0.0458 (11)	0.0455 (11)	−0.0047 (9)	0.0136 (9)	0.0041 (9)
C6	0.0557 (12)	0.0451 (11)	0.0470 (11)	−0.0009 (10)	0.0101 (10)	0.0065 (10)
C7	0.0498 (12)	0.0463 (12)	0.0521 (12)	0.0068 (9)	0.0099 (10)	0.0020 (10)
C8	0.0405 (11)	0.0498 (12)	0.0425 (11)	−0.0012 (9)	0.0095 (9)	−0.0013 (9)
C9	0.0396 (10)	0.0450 (11)	0.0394 (10)	−0.0053 (9)	0.0073 (9)	0.0018 (9)
C10	0.0454 (11)	0.0423 (11)	0.0477 (12)	0.0003 (9)	0.0125 (9)	0.0029 (9)
C11	0.0474 (12)	0.0601 (14)	0.0447 (11)	0.0037 (10)	0.0116 (10)	0.0042 (10)
C12	0.0441 (11)	0.0561 (13)	0.0543 (13)	0.0033 (10)	0.0117 (10)	0.0022 (10)
C13	0.0523 (13)	0.0434 (11)	0.0558 (13)	0.0006 (10)	0.0210 (11)	−0.0056 (10)
C14	0.0600 (14)	0.0581 (14)	0.0496 (12)	−0.0061 (11)	0.0147 (11)	−0.0129 (10)
C15	0.0481 (12)	0.0558 (13)	0.0533 (13)	−0.0051 (10)	0.0097 (10)	−0.0078 (10)
C16	0.0515 (13)	0.0772 (17)	0.0623 (14)	0.0151 (12)	0.0149 (11)	−0.0052 (13)
C17	0.0538 (14)	0.0707 (16)	0.0780 (17)	0.0002 (12)	0.0290 (12)	−0.0118 (13)

### Geometric parameters (Å, °)

**Table:**

O1—C8	1.360 (2)	C7—H7A	0.9300
O1—C16	1.434 (3)	C8—C9	1.433 (3)
O2—C13	1.373 (2)	C10—C11	1.390 (3)
O2—C17	1.429 (3)	C10—C15	1.396 (3)
N1—C1	1.313 (3)	C11—C12	1.388 (3)
N1—C9	1.371 (2)	C11—H11A	0.9300
C1—C2	1.401 (3)	C12—C13	1.378 (3)
C1—H1A	0.9300	C12—H12A	0.9300
C2—C3	1.374 (3)	C13—C14	1.390 (3)
C2—H2A	0.9300	C14—C15	1.379 (3)
C3—C4	1.430 (3)	C14—H14A	0.9300
C3—C10	1.490 (3)	C15—H15A	0.9300
C4—C9	1.419 (3)	C16—H16A	0.9600
C4—C5	1.420 (3)	C16—H16B	0.9600
C5—C6	1.358 (3)	C16—H16C	0.9600
C5—H5A	0.9300	C17—H17A	0.9600
C6—C7	1.409 (3)	C17—H17B	0.9600
C6—H6A	0.9300	C17—H17C	0.9600
C7—C8	1.369 (3)		
			
C8—O1—C16	117.66 (17)	C11—C10—C15	117.36 (19)
C13—O2—C17	118.02 (17)	C11—C10—C3	120.48 (18)
C1—N1—C9	116.31 (17)	C15—C10—C3	122.16 (18)
N1—C1—C2	124.90 (19)	C12—C11—C10	122.1 (2)
N1—C1—H1A	117.5	C12—C11—H11A	119.0
C2—C1—H1A	117.5	C10—C11—H11A	119.0
C3—C2—C1	120.3 (2)	C13—C12—C11	119.5 (2)
C3—C2—H2A	119.9	C13—C12—H12A	120.3
C1—C2—H2A	119.9	C11—C12—H12A	120.3
C2—C3—C4	117.12 (18)	O2—C13—C12	124.90 (19)
C2—C3—C10	121.17 (18)	O2—C13—C14	115.56 (19)
C4—C3—C10	121.69 (17)	C12—C13—C14	119.5 (2)
C9—C4—C5	119.13 (17)	C15—C14—C13	120.5 (2)
C9—C4—C3	118.05 (17)	C15—C14—H14A	119.7
C5—C4—C3	122.80 (18)	C13—C14—H14A	119.7
C6—C5—C4	120.22 (19)	C14—C15—C10	121.0 (2)
C6—C5—H5A	119.9	C14—C15—H15A	119.5
C4—C5—H5A	119.9	C10—C15—H15A	119.5
C5—C6—C7	121.45 (19)	O1—C16—H16A	109.5
C5—C6—H6A	119.3	O1—C16—H16B	109.5
C7—C6—H6A	119.3	H16A—C16—H16B	109.5
C8—C7—C6	120.08 (19)	O1—C16—H16C	109.5
C8—C7—H7A	120.0	H16A—C16—H16C	109.5
C6—C7—H7A	120.0	H16B—C16—H16C	109.5
O1—C8—C7	124.69 (18)	O2—C17—H17A	109.5
O1—C8—C9	115.14 (17)	O2—C17—H17B	109.5
C7—C8—C9	120.16 (18)	H17A—C17—H17B	109.5
N1—C9—C4	123.31 (18)	O2—C17—H17C	109.5
N1—C9—C8	117.77 (17)	H17A—C17—H17C	109.5
C4—C9—C8	118.92 (17)	H17B—C17—H17C	109.5

### Hydrogen-bond geometry (Å, °)

**Table:**

Cg2and Cg3 are the centroids of the C4–C9 and C10–C14 rings, respectively.

**Table:**

*D*—H···*A*	*D*—H	H···*A*	*D*···*A*	*D*—H···*A*
C17—H17A···O1^i^	0.96	2.55	3.322 (3)	137
C2—H2A···Cg2^ii^	0.93	2.81	3.622 (2)	146
C6—H6A···Cg3^iii^	0.93	2.80	3.592 (2)	144
C17—H17B···Cg2^iv^	0.96	2.78	3.580 (3)	142

Symmetry codes: (i) *x*−1, −*y*+1/2, *z*+1/2; (ii) −*x*, *y*+1/2, −*z*+1/2; (iii) −*x*, −*y*, −*z*+1; (iv) *x*−1, *y*, *z*.
